# Antiretrovirals Promote Metabolic Syndrome through Mitochondrial Stress and Dysfunction: An In Vitro Study

**DOI:** 10.3390/biology12040580

**Published:** 2023-04-10

**Authors:** Jivanka Mohan, Terisha Ghazi, Thabani Sibiya, Anil A. Chuturgoon

**Affiliations:** Discipline of Medical Biochemistry, School of Laboratory Medicine and Medical Sciences, University of KwaZulu-Natal, Durban 4041, South Africa

**Keywords:** metabolic syndrome, ARVs, mitochondrial stress, mitochondrial dysfunction, oxidative stress, insulin resistance

## Abstract

**Simple Summary:**

Antiretrovirals have several side effects. Recently, the use of antiretrovirals has been associated with metabolic syndrome. Metabolic syndrome arises through several abnormalities including mitochondrial dysfunction. The current study aimed to ascertain the effects of singular and combinational usage of current ARVs on mitochondrial dysfunction and its linkage to metabolic syndrome in liver (HepG2) cells. Cells were treated for a period of 120 h using Tenofovir disoproxil fumarate, Lamivudine, and Dolutegravir in singular and combinational doses. Results indicated that ARVs induce mitochondrial stress and dysfunction, which may be closely associated with the progression of insulin resistance. The evidence can be used to develop therapies with reduced side effects related to metabolic syndrome.

**Abstract:**

The prevalence of metabolic syndrome MetS in HIV-infected patients on chronic antiretroviral (ARV) therapy continues to rise rapidly, with an estimated 21% experiencing insulin resistance. The progression of insulin resistance is strongly related to mitochondrial stress and dysfunction. This study aimed to draw links between the singular and combinational use of Tenofovir disoproxil fumarate (TDF), Lamivudine (3TC), and Dolutegravir (DTG) on mitochondrial stress and dysfunction as an underlying mechanism for insulin resistance following a 120 h treatment period using an *in vitro* system of human liver cells (HepG2). The relative protein expressions of pNrf2, SOD2, CAT, PINK1, p62, SIRT3, and UCP2, were determined using Western blot. Transcript levels of PINK1 and p62 were assessed using quantitative PCR (qPCR). ATP concentrations were quantified using luminometry, and oxidative damage (malondialdehyde (MDA) concentration) was measured using spectrophotometry. The findings suggest that despite the activation of antioxidant responses (pNrf2, SOD2, CAT) and mitochondrial maintenance systems (PINK1 and p62) in selected singular and combinational treatments with ARVs, oxidative damage and reduced ATP production persisted. This was attributed to a significant suppression in mitochondrial stress responses SIRT3 and UCP2 for all treatments. Notable results were observed for combinational treatments with significant increases in pNrf2 (*p* = 0.0090), SOD2 (*p* = 0.0005), CAT (*p* = 0.0002), PINK1 (*p* = 0.0064), and p62 (*p* = 0.0228); followed by significant decreases in SIRT3 (*p* = 0.0003) and UCP2 (*p* = 0.0119) protein expression. Overall there were elevated levels of MDA (*p* = 0.0066) and decreased ATP production (*p* = 0.0017). In conclusion, ARVs induce mitochondrial stress and dysfunction, which may be closely associated with the progression of insulin resistance.

## 1. Introduction

Metabolic syndrome (MetS) is a non-communicable disease affecting 20–30% of adults worldwide. Due to the increased incidence of MetS over the years, the World Health Organization (WHO) has classified the cluster of pathologies as a global hazard. The pathologies that can occur include hypertension, insulin resistance, and dyslipidaemia [1,2]. The occurrence of one or more of the pathologies can result in severe diseases such as cardiovascular diseases and Type 2 Diabetes Mellitus (T2DM).

The occurrence of MetS in people living with HIV (PLWH) has been described extensively in previous studies [3]. HIV affects the global population; however, the most severe effects and prevalence are observed in Sub-Saharan Africa. By the end of 2019, roughly 38 million cases of HIV were reported worldwide, with 7.8 million cases being localised to South Africa [4,5].

Of the infected population, 26 million had access to ARV treatment [4]. Highly active antiretroviral therapy (HAART) has been associated with a significant decrease in the mortality rate in PLWH [6]. However, the other side effects remain numerous, with clinical studies showing a correlation between HAART usage and MetS. At least 21% of PLWH using HAART displayed insulin resistance [7,8,9]. Interestingly, the prevalence of T2DM and hyperglycaemia is higher in HIV positive patients using ARVs as opposed to the general population [10]. Despite the severity and rising incidence of cases of MetS following HAART use, very few biochemical studies exist showing the mechanisms of action in MetS promotion following ARV usage.

One of the most common biochemical outcomes observed in MetS is mitochondrial dysfunction. It is well understood that mitochondrial stress contributes to reactive oxygen species (ROS) production, oxidative stress, and inflammation, which are strongly associated with MetS [1]. Previous evidence links mitochondrial dysfunction and inflammation into an underlying process that can promote insulin resistance.

Several different pathways and proteins ameliorate mitochondrial stress. Two of the most common proteins involved in mitochondrial stress maintenance are Sirtuin 3 (SIRT3) and Uncoupling protein 2 (UCP2) [11]. Under oxidative stress conditions, UCP2 is activated to increase NAD+/NADH ratios in cells to lead to the activation of SIRT3 [1]. SIRT3 functions by deacetylating other regulatory proteins, such as superoxide dismutase 2 (SOD2) and catalase (CAT) [12]. However, the activation of such proteins is not exclusive to SIRT3 activity but may occur through upregulation of the transcription factor nuclear factor (erythroid-derived 2)-like 2 (Nrf2) [13,14]. Nrf2 is responsible for the transcription of several genes involved in a cell’s antioxidant response [15].

Additionally, Nrf2 has been highlighted in the activation of mitochondrial maintenance genes/proteins [16]. PTEN-induced kinase 1 (PINK1) and ubiquitin-binding protein p62 (p62) are both activated in response to oxidative stress. PINK1 deficiency has been associated with excess ROS production and aberrant mitochondrial respiration [17]. On the other hand, p62 is upregulated in response to protein damage as a method of removal of dysfunctional proteins [18]. Nrf2 transcriptionally regulates PINK1 and p62 during oxidative stress [19,20]. Mitochondrial dysfunction and subsequent excess ROS production promote insulin resistance by activating c-Jun N-terminal kinases (JNK) and NLR family pyrin domain containing 3 (NLRP3) inflammasome which leads to the deactivation of the IRS1/PI3K/AKT pathway and the progression of insulin resistance [21].

Insulin signalling is imperative for glucose uptake in cells; however, in the liver, insulin is responsible for the initiation of fatty acid synthesis through the regulation of de novo lipogenesis. Aberrations lead to fatty acid accumulation and progression of non-alcoholic fatty liver disease (NAFLD) [22]. The latter is closely linked with the progression in T2DM [23].

In 2016, the WHO proposed the usage of TDF, 3TC, and Emtricitabine (FTC)/Efavirenz (EFV) as the preferred combinational treatment for HIV in young adolescents and adults. However, as research progressed, the WHO updated its recommendations. In 2018, DTG was recommended for usage in first-line treatment, leading to combinational treatment of 3TC, TDF, and DTG being popularised following approval by the WHO [24].

Studies often assess the side effects of these drugs in isolation, with very few studies evaluating biochemical mechanisms involved in their combinational usage [25]. This study aimed to ascertain the relationship between the singular and combinational use of the drugs in mitochondrial stress and dysfunction in vitro as a possible method of MetS promotion. In order to achieve the aims, a HepG2 cell model was used for testing. These liver cells are commonly used for in vitro experiments involving drugs as they display similar physiological functions and genetic profiles as primary hepatocytes. [26,27,28]. Furthermore, HepG2 cells have been used to test antiretroviral toxicity with specificity to mitochondrial and oxidative stress [27,29]. An exposure period of 120 h was used for testing in line with other studies using ARVs and HepG2 cells [27,28]. Evidence from the current study can be used to develop therapies with reduced side effects related to MetS.

## 2. Materials and Methods

Antiretroviral drugs (3TC, TDF, and DTG) were sourced from the NIH AIDS reagents program. HepG2 cells were obtained from American Type Culture Collection (Johannesburg, South Africa). Cell culture reagents were purchased from Lonza (Basel, Switzerland). Luminometry kits (ATP) were obtained from Promega (Madison, WI, USA). Materials for Western Blots were obtained from Bio-Rad (Hercules, CA, USA). The remaining reagents were obtained from Merck (Darmstadt, Germany) unless stated otherwise.

### 2.1. Cell Culture and Treatment

Culturing of HepG2 cells was carried out in 25 cm^3^ cell culture flasks using complete culture medium (CCM) (Eagle’s minimum essentials medium (EMEM) supplemented with 10% foetal calf serum, 1% pen-strep-fungizone, and 1% L-glutamine) and incubated in a humidified incubator (37 °C, 5% CO_2_) until roughly 80% confluency was reached. Thereafter cells were treated with the physiological concentrations (C_max_—maximum plasma concentration) of ARVs (3TC: 1.51 µg/mL, TDF: 0.3 µg/mL, DTG: 3.67 µg/mL) [19,20,21] for 120 h (h) as per Nagiah et al., 2015 [27]. ARVs were dissolved in phosphate-buffered saline (PBS) for treatment. Fresh media and ARVs were replenished every 24 h. All subsequent assays were carried out following treatment as explained above. Untreated cells containing CCM only were used for controls.

### 2.2. ATP Quantification

ATP quantification was assessed using the CellTiter-Glo^®^ Luminescent Cell Viability Assay (Promega, Madison, WI, USA, #G7570). The assay was performed as per the manufacturer’s instructions. Luminescence was measured using a Modulus™ Microplate Reader (Turner Biosystems, Sunnyvale, CA, USA). Results are expressed as relative light units (RLU).

### 2.3. Lipid Peroxidation-TBARS Assay

Oxidative damage was assessed using the thiobarbituric acid assay, which quantifies the malondialdehyde (MDA) levels, a by-product of lipid peroxidation. MDA concentration is proportional to ROS production in cells. Following treatment, the supernatant was collected from flasks and added to test tubes (200 μL). This was supplemented with 2% H_3_PO_4_ (200 μL), 7% H_3_PO_4_ (200 μL), and thiobarbituric acid/butylated hydroxytoluene solution (400 μL). The pH of all samples was adjusted to 1.5 and then they were boiled for 15 min. After cooling, samples were supplemented with 1.5 mL of butanol and vortexed for separation into distinct phases. Following separation, 100 μL of the upper phase of each sample was dispensed into a 96-well microtitre plate in triplicates. The optical density was measured on a spectrophotometer at 532 nm with a reference wavelength of 600 nm. The average optical density was calculated and divided by the absorption coefficient (156 mM^−1^). Results were represented as MDA concentration (μM).

### 2.4. Western Blots

Following 120 h of treatment, protein was isolated and standardised as per Sibiya et al., 2022 [29].

Thereafter, Western blots were performed as per Nagiah et al., 2015 [27].

Primary antibodies (1:1000 in 5% BSA) were using for probing (Table 1). Following this, membranes were washed (5 × 10 min in TTBS) and HRP-conjugated secondary antibodies were added to membranes for 1 h at RT (Cell signalling Technology; anti-rabbit (#7074S); anti-mouse (#7076S) 1:5000 in 5% BSA). Following incubation, membranes were washed (5 × 10 min in TTBS) and rinsed with distilled water. Protein detection was carried out using the Clarity Western ECL Substrate detection reagent (400 µL) (Bio-Rad, Hercules, CA, USA), and images were captured using the Bio-Rad ChemiDoc™ XRS+ Imaging System. The same membranes were used for multiple proteins. This was achieved by stripping membranes using 5% hydrogen peroxide for 30 min at 37 °C, blocking with 5% BSA, and re-probing using new antibodies.

Once all target protein detections were completed, membranes were stripped and incubated with HRP-conjugated antibody for β-actin (A3854, Sigma-Aldrich, St. Louis, MO, USA). β-actin is a housekeeping protein expressed evenly across cells. Image Lab™ Software v6.0 (Bio-Rad, Hercules, CA, USA) was used to analyse the results. The relative band density of protein was calculated by normalising results against β-actin.

### 2.5. Quantitative PCR

#### 2.5.1. RNA Isolation and Quantification

Following treatment, RNA isolation and standardisation was performed as per Sibiya et al., 2022 [29].

#### 2.5.2. Quantification of mRNA Expression

Following standardisation, cDNA was synthesised using the iScript™ cDNA Synthesis kit as per the manufacturer’s instructions (Bio-Rad, 107-8890, Hercules, CA, USA).

Transcript levels of relevant genes (Table 2) were assessed using the CFX96 Touch™ Real-Time PCR Detection System (Bio-Rad, Hercules, CA, USA) and SsoAdvanced™ Universal SYBR^®^ Green Supermix (Bio-Rad, 1725270). The thermo-cycler conditions for each gene were as follows: initial denaturation (8 min, 95 °C), followed by 40 cycles of denaturation (15 s, 95 °C), annealing (40 s, Table 1), and extension (30 s, 72 °C). GAPDH is evenly expressed across cells and was used for normalisation. Results were calculated using the Livak and Schmittgen (2001) method and were represented as fold change relative to the control cells (2^−ΔΔCT^) [30].

### 2.6. Statistical Analysis

GraphPad Prism version 5.0 (GraphPad Software Inc., San Diego, CA, USA) was used to perform all statistical analyses. Data were analysed using an unpaired *t*-test (control vs. treatment) and represented as the mean ± standard deviation unless otherwise stated. A value of *p* ˂ 0.05 was considered statistically significant.

## 3. Results

### 3.1. Antiretrovirals Activated Antioxidant Responses in HepG2 Cells

**Figure 1:**
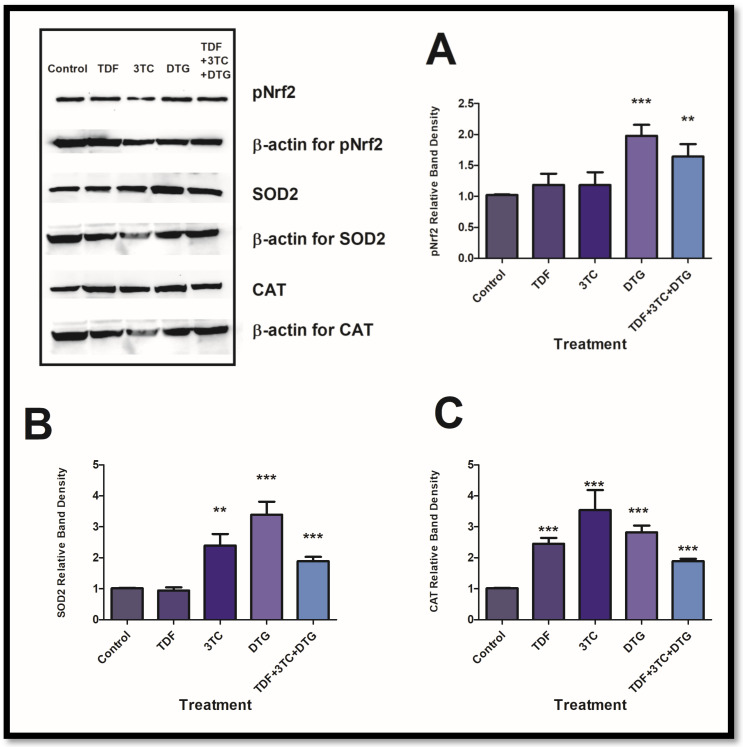
Antiretrovirals activated antioxidant responses. Protein expression of pNrf2 (**A**) and SOD2 (**B**) was significantly increased in selected treatments, whereas all treatments increased protein expression of CAT (**C**). ** *p* < 0.005, *** *p* < 0.0001 relative to control (Raw blot images are available in Supplementary Materials: Figures S1–S12).

Phosphorylated Nrf2 (Ser40) (pNrf2) is the active form of Nrf2 that can translocate to the nucleus and transcribe for proteins. It is activated under oxidative stress conditions. Following treatment with ARVs, only DTG (*p* = 0.0008) and combinational treatments (*p* = 0.0059) showed significant increases in pNrf2 expression (Figure 1A). Significant increases in protein expression of SOD2 were only observed for 3TC (*p* = 0.0029), DTG (*p* = 0.0006), and combinational usage (*p* = 0.0004) (Figure 1B). All treatments were able to increase the protein expression of CAT (TDF; (*p* = 0.0002), 3TC; (*p* = 0.0025), DTG; (*p* = 0.0001), combinational use; (*p* < 0.0001)) (Figure 1C).

### 3.2. Antiretrovirals Activated Mitochondrial Maintenance Intermediates

**Figure 2:**
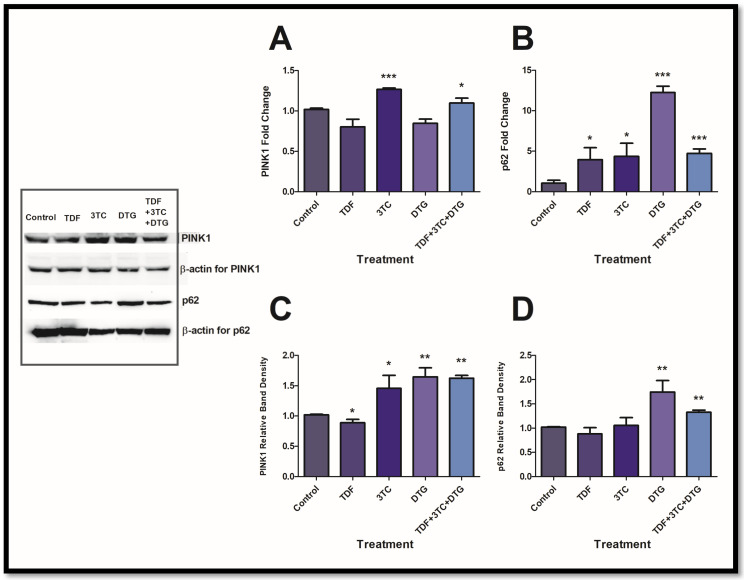
Transcript and protein expression of mitochondrial maintenance components. PINK1 mRNA expression (**A**) and protein expression (**C**) as elevated for selected ARV treatments. Significant increases in *p62* mRNA expression were observed for all treatments (**B**); however, only DTG and combinational usage showed considerable increases in p62 protein expression (**D**). * *p* < 0.05, ** *p* < 0.005, *** *p* < 0.0001 relative to control (Raw blot images are available in Supplementary Materials: Figures S1–S12).

*PINK1* gene expression (Figure 2A) increased significantly with 3TC (*p* = 0.0002) and combinational treatments (*p* = 0.0383); however, protein expression was elevated by 3TC (*p* = 0.0246), DTG (*p* = 0.0019), and combinational treatments (*p* = 0.0001) (Figure 2C). Interestingly, *p62* mRNA expression (Figure 2B) was elevated for all treatments (TDF; (*p* = 0.0298), 3TC; (*p* = 0.0259), DTG; (*p* = 0.0002), combinational use; (*p* = 0.0006)); however, protein expression only showed significant elevations in DTG (*p* = 0.0061) and combinational usage treatments (*p* = 0.0011) (Figure 2D).

### 3.3. Antiretrovirals Suppress Essential Mitochondrial Stress Proteins

**Figure 3:**
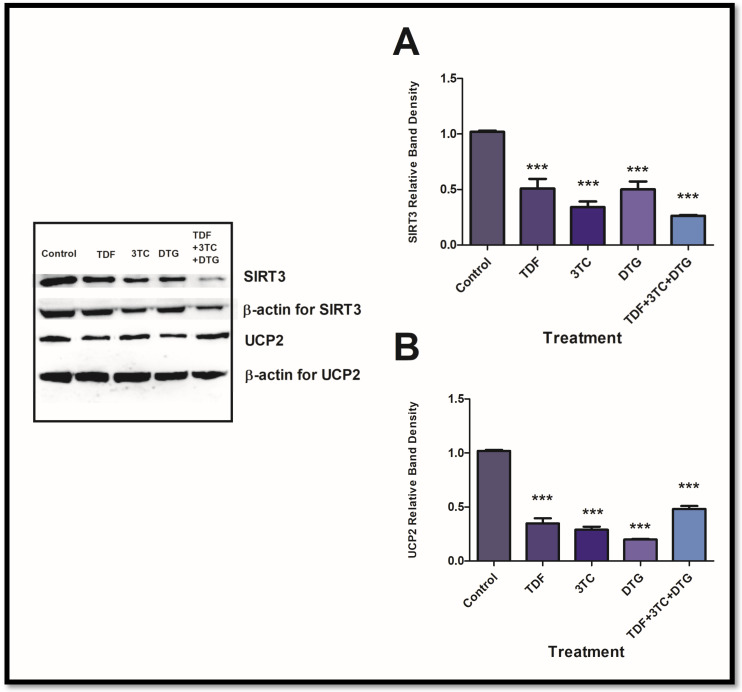
ARVs suppressed mitochondrial stress responses. SIRT3 protein expression (**A**) was significantly decreased with corresponding decreases in UCP2 protein expression (**B**). *** *p* < 0.0001 relative to control (Raw blot images are available in Supplementary Materials: Figures S1–S12).

SIRT3 is a crucial regulator of mitochondrial stress, and significant decreases in protein expression were observed for all treatments (TDF; (*p* = 0.0005), 3TC; (*p* < 0.001), DTG; (*p* = 0.0002), combinational use; (*p* < 0.0001)) (Figure 3A). This was followed by coinciding decreases in UCP2 protein expression (TDF; (*p* =< 0.0001), 3TC; (*p* < 0.0001), DTG; (*p* < 0.0001), combinational use; (*p* < 0.0001)) (Figure 3B), indicating a suppression in mitochondrial stress responses.

### 3.4. Antiretrovirals Increased Lipid Peroxidation and Decreased ATP Concentrations

**Figure 4:**
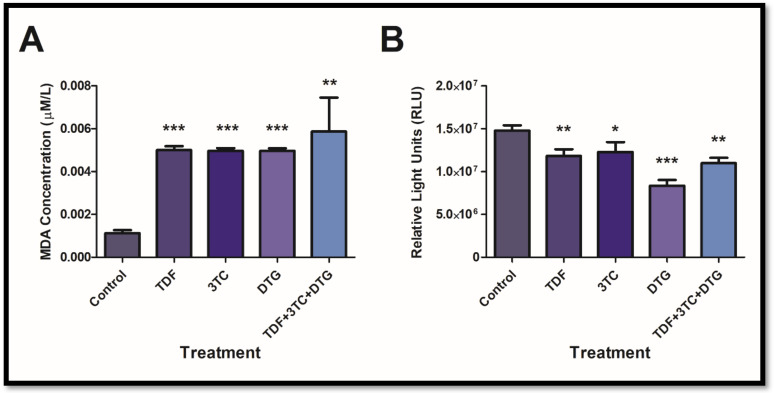
ARVs increased MDA concentration (**A**) and decreased ATP concentration (**B**). Considerable increases in MDA were observed for all ARV treatments (**A**), with significant decreases in ATP. * *p* < 0.05, ** *p* < 0.005, *** *p* < 0.0001 relative to control.

MDA concentration is indicative of lipid peroxidation in cells and is proportional to ROS formation in cells. Singular treatments, TDF (*p* < 0.0001), 3TC (*p* < 0.0001), and DTG (*p* < 0.0001), showed significant increases in MDA concentration (Figure 4A). This was further observed in the combinational treatment (*p* = 0.0066) (Figure 4A). ATP concentrations are used to assess mitochondrial function where decreases indicate compromised mitochondrial function. All singular treatments, TDF (*p* = 0.0065), 3TC (*p* = 0.0299), and DTG (*p* = 0.0003), showed significant reductions in ATP concentration with similar results observed for combinational treatment (*p* = 0.0017) (Figure 4B).

## 4. Discussion

MetS from chronic drug use is induced when one or more metabolic irregularities occur in the human body. One of the most common pathologies associated with MetS is insulin resistance, which can lead to the pathogenesis of T2DM if not controlled [31,32]. Several different biochemical abnormalities may cause insulin resistance; however, mitochondrial stress and dysfunction have been highlighted as one of the most frequent causes of insulin resistance [33]. Mitochondrial stress leads to aberrations in the electron transport chain (ETC), resulting in decreased ATP production and leakage of ROS [33]. Increased ROS production and leakage are responsible for activating the NLRP3 inflammasome [34,35] and the upregulation of JNK [21]. These processes result in increased phosphorylation of the insulin receptor substrate 1 (IRS1) and subsequent decreased activity of Protein kinase B (AKT) and phosphoinositide 3-kinase (PI3K). Consequently, reduced glucose uptake, vasodilation, and insulin secretion are experienced, resulting in the progression of insulin resistance [21].

Alarmingly, the prevalence of MetS in PLWH and ARV usage is increasing rapidly, with at least 21% experiencing insulin resistance [7,8,9]. Newer generation ARVs are associated with fewer side effects than older generations; however, metabolic complications persist with usage [6,24]. This study determined the impact of singular and combinational use of ARVs on mitochondrial stress as an underlying mechanism for insulin resistance promotion.

Nrf2 has been described extensively for its endogenous role in antioxidant responses. It allows for the transcriptional activation of several genes required to ameliorate oxidative stress [15]. Nrf2 can lead to the activation of SOD_2_ and CAT; however, the latter two enzymes can also be independently activated in response to high levels of ROS and inflammation [36,37]. SOD_2_ is located in the mitochondrial matrix, making it a suitable indicator for mitochondrial stress [38]. In the present study, only DTG and combinational usage significantly increased the expression of pNrf2 (Figure 1A). However, it is important to note that the remaining treatments did not decrease pNrf2 expression, but rather showed no significant changes. Previous studies in HepG2 cells using older generation ARVs showed selective upregulation of Nrf2 [27], and support the results from the current study. The significant upregulation of pNrf2 following combinational usage indicates possible oxidative stress.

Interestingly, SOD_2_ protein expression was increased by 3TC, DTG, and combinational usage (Figure 1B), suggesting that high levels of ROS were present in the mitochondria following exposure. This was followed by an increase in CAT expression (Figure 1C). The results correlate with ambient and upregulated pNrf2 expression. Previous studies have shown strong links between DTG usage and increased ROS production following the deregulation of Ca_2+_ signalling [39]. Increased ROS potential via DTG exposure coincides with upregulated responses in this study.

Although only selected singular treatments were able to upregulate antioxidant responses, the significant increase in combinational treatment indicates synergistic stress induced by combining different ARVs. This is critical information as ARVs are rarely ingested individually, instead, they are in a single-dose tablet that contains all three drugs.

PINK1 and p62 expressions were next analysed due to the prevailing oxidative stress environment. PINK1 and p62 are mitochondrial maintenance mediators that can be activated by Nrf2 [19,20]. PINK1 is upregulated to ensure mitochondrial respiration occurs and ROS production is reduced [17]. Although only 3TC and combinational treatments increased PINK1 mRNA levels (Figure 2A), the protein expression of PINK1 for 3TC, DTG, and combinational drug treatments was upregulated (Figure 2C). Following treatment with these ARVs, PINK1 was possibly post-transcriptionally upregulated in response to aberrations in mitochondrial function. The results agree with increases in pNrf2 expression for selected treatments.

Conversely, we observed significant elevations in transcript levels of p62, but significant increases were only noted for DTG and combinational ARV usage of p62 protein expression (Figure 2B,D). Literature indicates that p62 is upregulated in response to protein damage as a method for the clearance of oxidatively damaged proteins [18]. This suggests that DTG and combinational ARV treatments induced damage of proteins, thus eliciting significant upregulation in p62 expression. Additionally, p62 can activate Nrf2 in a positive feedback loop in response to oxidative stress in cells [19,40]. The considerable upregulation in pNrf2 and p62 for DTG and combinational ARV treatment suggests that the positive feedback loop was activated in response to stress in the HepG2 cells. Moreover, no significant changes in pNrf2 and p62 protein expression were observed for TDF and 3TC treatments, suggesting possible absence of the loop.

All treatments with ARVs showed significant downregulation in SIRT3 and UCP2 expression (Figure 3). Both proteins play a role in the amelioration of mitochondrial stress. More specifically, suppression of SIRT3 has strongly been associated with mitotoxicity due to inadequate stress relief. UCP2 is known to activate SIRT3, among other stress responses in cells which are imperative to mitochondrial integrity and function [11]. Furthermore, SIRT3 is responsible for the deacetylation of complexes in the ETC, which maintains ATP production, while UCP2 is responsible for oxygen consumption in ATP synthesis [41]. Aside from this, UCP2 has been associated with reductions in mitochondrial oxidative stress, and dysfunction in the protein has been linked to cardiovascular diseases [42]. It can be deduced that aberrations in mitochondrial stress responses were experienced following exposure to all drugs. Additionally, SOD2 and CAT were activated independently of SIRT3 activity.

Following exposure to ARVs, oxidative damage occurred in the form of lipid peroxidation, which has previously been associated with ROS production [43]. MDA concentration (Figure 4A) was significantly upregulated despite the activation of antioxidant responses and mitochondrial maintenance in selected treatments. Older generation ARVs showed increased MDA levels in HepG2 cells [23], indicating that newer-generation ARVs have similar adverse effects. Furthermore, usage of TDF and 3TC increased lipid peroxidation and depleted glutathione levels when paired with Efavirenz in rat liver and kidneys [44]. The increase in oxidative damage indicates that the cells had insufficient antioxidant responses.

**Figure A1:**
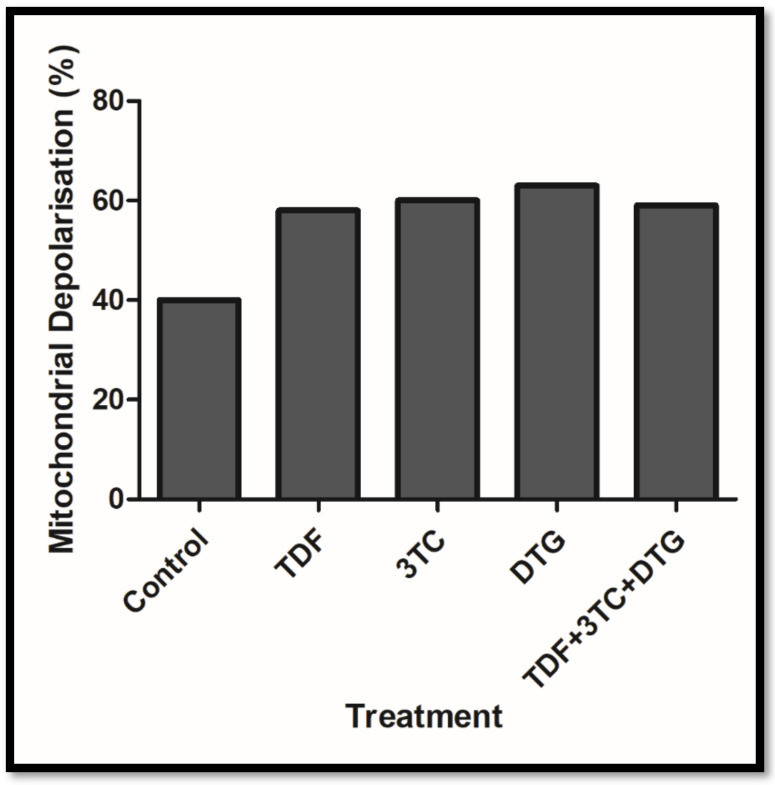
Percentage of mitochondrial depolarisation following treatment. All treatments showed an increase in depolarisation however values were not significant.

Significant reductions in ATP production for all treatments were observed (Figure 4B). The considerable suppression in mitochondrial stress proteins SIRT3 and UCP2 (Figure 3) provides reasoning for decreased ATP production despite PINK1 upregulation (Figure 2C). Reduced ATP production is indicative of dysfunctional mitochondrial activity [45]. Previous studies have shown that DTG and TDF decreased mitochondrial ATP production via action on the ETC [46,47], supporting the findings in the present study. We further observed increased depolarisation of mitochondrial membranes, which is indicative of dysfunction; however, results were not significant (Appendix A Figure A1).

Increased oxidative damage is a sign of excess ROS production, which coincides with decreased ATP production. The current study provides evidence that ROS production increased despite an attempt in liver cells to upregulate antioxidant responses. This was mainly attributed to suppressed mitochondrial stress responses. Elevations in mitochondrial ROS have been shown to activate JNK and the NLRP3 inflammasome, both of which are responsible for the phosphorylation of IRS1 through several intermediates. Subsequently, insulin resistance can be promoted through decreased action of the IRS1/PI3K/AKT pathway [21]. The present study, therefore, provides evidence that combinational usage of ARVs can lead to mitochondrial dysfunction that can promote insulin resistance. This information is crucial as HAART is popularly consumed in combination.

## 5. Future Recommendations and Limitations

The present study was an in vitro study, which has limitations in terms of application to humans. Future studies should assess similar markers in an in vivo humanised HIV+ mouse model to fully understand the mechanism of ARV induction of MetS. Aside from this, different exposure periods should be considered. Furthermore, studies assessing effects on the IRS1/PI3K/AKT axis and inflammasome activation are recommended to enhance findings.

## Figures and Tables

**Table 1:** Antibodies used for immunoprobing.

Antibody	Company	Catalogue Number
Anti-PINK1 antibody [N4/15]	Abcam	ab186303
Anti-SIRT3	Abcam	ab264041
Recombinant Anti-Nrf2 (phospho S40) antibody [EP1809Y]	Abcam	ab76026
Anti-SQSTM1/p62 antibody [2C11]—BSA and Azide free	Abcam	ab56416
SOD2 (D9V9C) Rabbit mAb	Cell Signalling	13194S
Catalase (D4P7B) Rabbit mAb	Cell Signalling	12980S
UCP2 (D1O5V) Rabbit mAb	Cell Signalling	89326S

**Table 2:** Primer sequences with respective annealing temperatures for genes assessed.

Gene		Sequence (5′-3′)	Annealing Temperature (°C)
*PINK1*	Forward Reverse	GGAGGAGTATCTGATAGGGCAG AACCCGGTGCTCTTTGTCAC	57
*p62*	Forward Reverse	CAGAGAAGCCCATGGACAG AGCTGCCTTGTACCCACATC	60
*GAPDH*	Forward Reverse	TCCACCACCCTGTTGCTGTA ACCACAGTCCATGCCATCAC	---

## Data Availability

Not applicable.

## Supplementary Materials

The following supporting information can be downloaded at https://www.mdpi.com/article/10.3390/biology12040580/s1, Figure S1. Western blot membrane of pNrf2 (~105 kDa); Figure S2. Western blot membrane of p62 (~62 kDa); Figure S3. Western blot membrane of Beta-Actin (~42 kDa); Figure S4. Western blot membrane of CAT (~57 kDa); Figure S5. Western blot membrane of SOD2 (~25 kDa); Figure S6. Western blot membrane of Beta-Actin for SOD2 and Catalase (~42 kDa); Figure S7. Western blot membrane of UCP2 (~33 kDa); Figure S8. Western blot membrane of Beta-Actin for UCP2 (~42 kDa); Figure S9. Western blot membrane of SIRT3 (~44 kDa); Figure S10. Western blot membrane of Beta-Actin for SIRT3 (~42 kDa); Figure S11. Western blot membrane of PINK1 (~63 kDa); Figure S12. Western blot membrane of Beta-Actin for PINK1 (~42 kDa).
