# Supplementary material for: Antiretrovirals Promote Metabolic Syndrome through Mitochondrial Stress and Dysfunction: An In Vitro Study
Source: Biology (Basel). 2023 Apr 10;12(4):580. doi: 10.3390/biology12040580 (PMC10135454; doi:10.3390/biology12040580)
Supplement: Supplementary file 1 [file biology-12-00580-s001.zip › biology-2283835-supplementary.pdf]

Supplementary materials

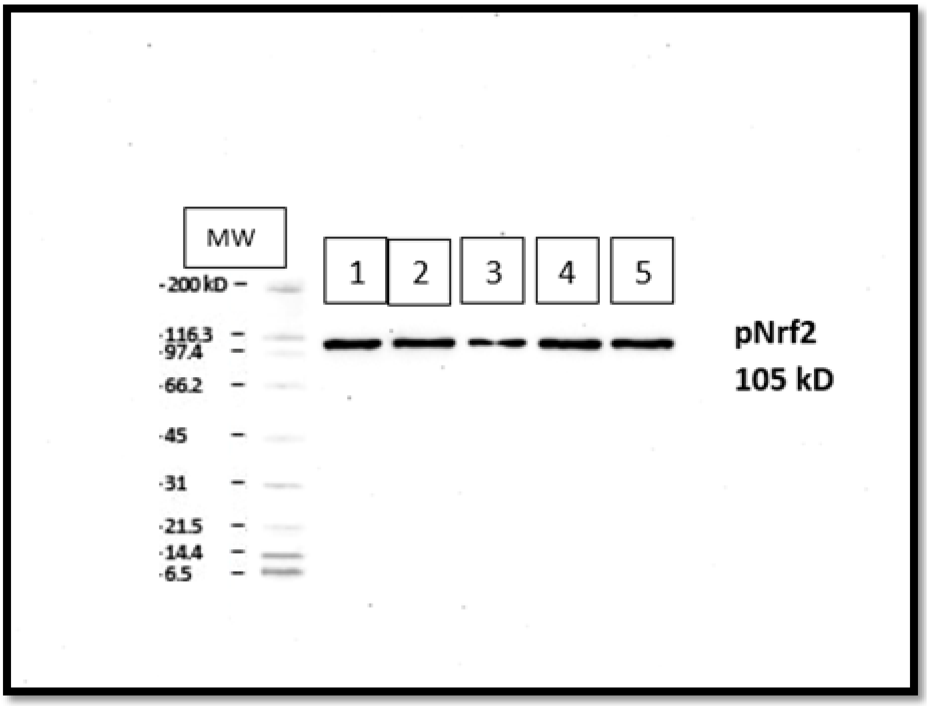

Figure S1. Western blot membrane of pNrf2 (~105 kDa). Protein detected with Recombinant Anti-Nrf2 (phospho S40) antibody [EP1809Y] (Abcam; ab76026). Membranes were incubated with HRP-conjugated secondary antibody (Cell signalling Technology; anti-rabbit (#7074S)). Densitometry readings were calculated using Image Lab™ Software v6.0 (Bio-Rad, Hercules, CA, USA). ([MW-Molecular Weight marker]; [1- Control]; [2- TDF]; [3- 3TC]; [4- DTG]; [5- TDF+3TC+DTG]).

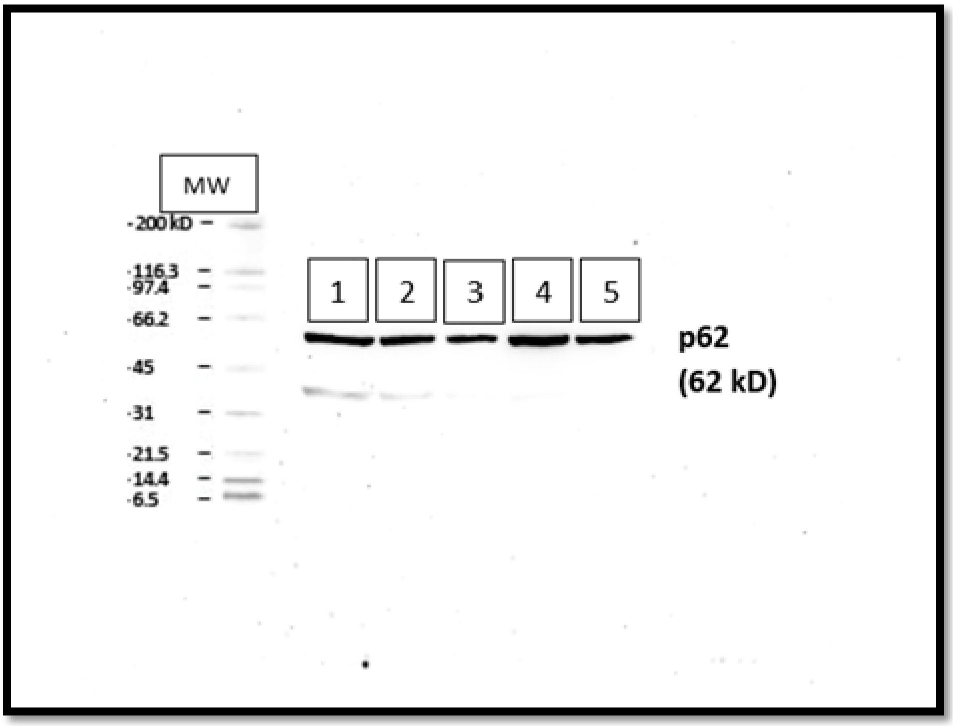

Figure S2. Western blot membrane of p62 (~62kDa). Protein detected with Anti-SQSTM1 / p62 antibody [2C11] - BSA and Azide free (Abcam; ab56416). Membranes were incubated with HRP-conjugated secondary antibody (Cell signalling Technology; anti-mouse (#7076S)). Densitometry readings were calculated using Image Lab™ Software v6.0 (Bio-Rad, Hercules, CA, USA). ([MW-Molecular Weight marker]; [1- Control]; [2- TDF]; [3- 3TC]; [4- DTG]; [5- TDF+3TC+DTG])

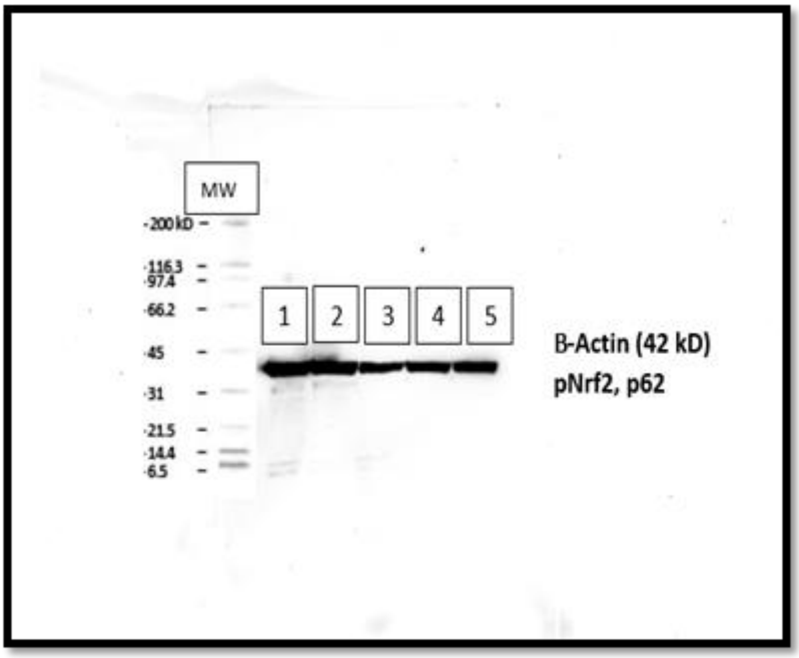

Figure S3. Western blot membrane of Beta-Actin (~42 kDa). Protein detected with HRP-conjugated antibody for β-actin (A3854, Sigma-Aldrich). Densitometry readings were calculated using Image Lab™ Software v6.0 (Bio-Rad, Hercules, CA, USA). ([MW-Molecular Weight marker]; [1- Control]; [2- TDF]; [3- 3TC]; [4- DTG]; [5- TDF+3TC+DTG]).

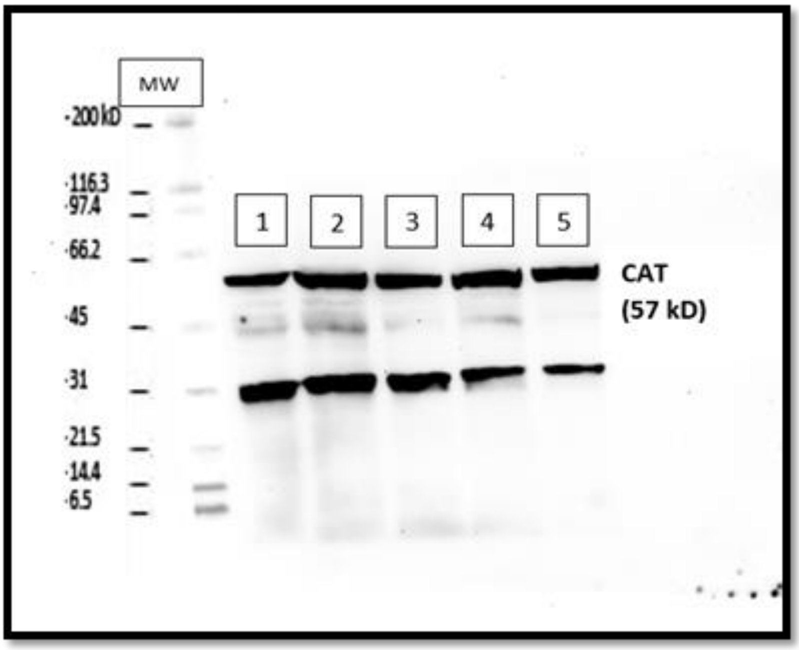

Figure S4. Western blot membrane of CAT (~57 kDa). Protein detected with Catalase (D4P7B) Rabbit mAb (Cell signalling Technology; #12980S). Membranes were incubated with HRP-conjugated secondary antibody (Cell signalling Technology; anti-rabbit (#7074S)). Densitometry readings were calculated using Image Lab™ Software v6.0 (Bio-Rad, Hercules, CA, USA). ([MW-Molecular Weight marker]; [1- Control]; [2- TDF]; [3- 3TC]; [4- DTG]; [5- TDF+3TC+DTG])

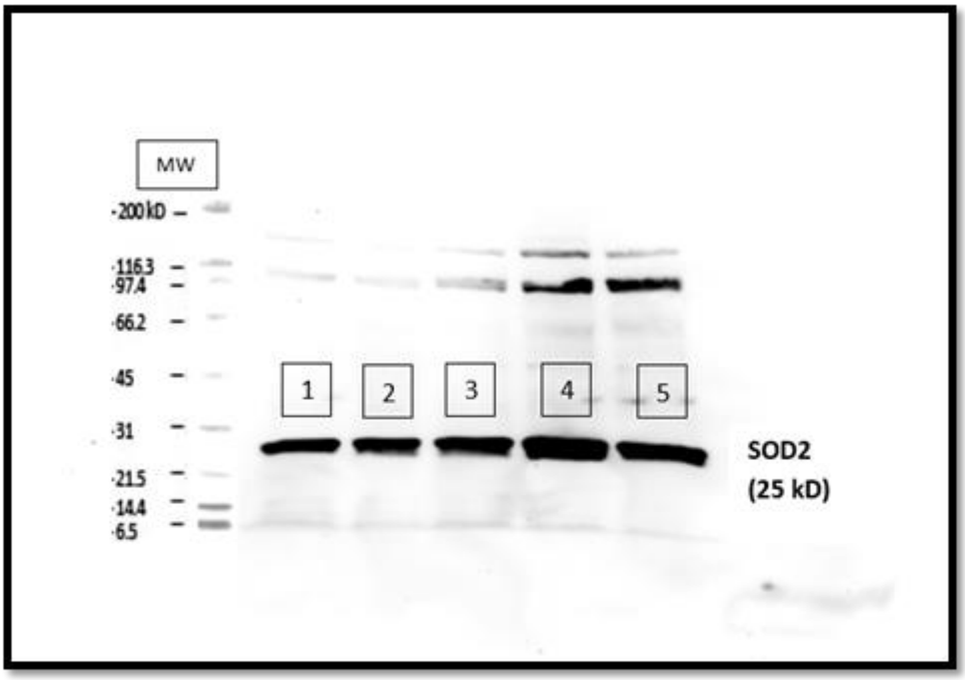

Figure S5. Western blot membrane of SOD2 (~25 kDa). Protein detected with SOD2 (D9V9C) Rabbit mAb (Cell signalling Technology; #13194S). Membranes were incubated with HRP-conjugated secondary antibody (Cell signalling Technology; anti-rabbit (#7074S)). Densitometry readings were calculated using Image Lab™ Software v6.0 (Bio-Rad, Hercules, CA, USA). ([MW-Molecular Weight marker]; [1- Control]; [2- TDF]; [3- 3TC]; [4- DTG]; [5- TDF+3TC+DTG]).

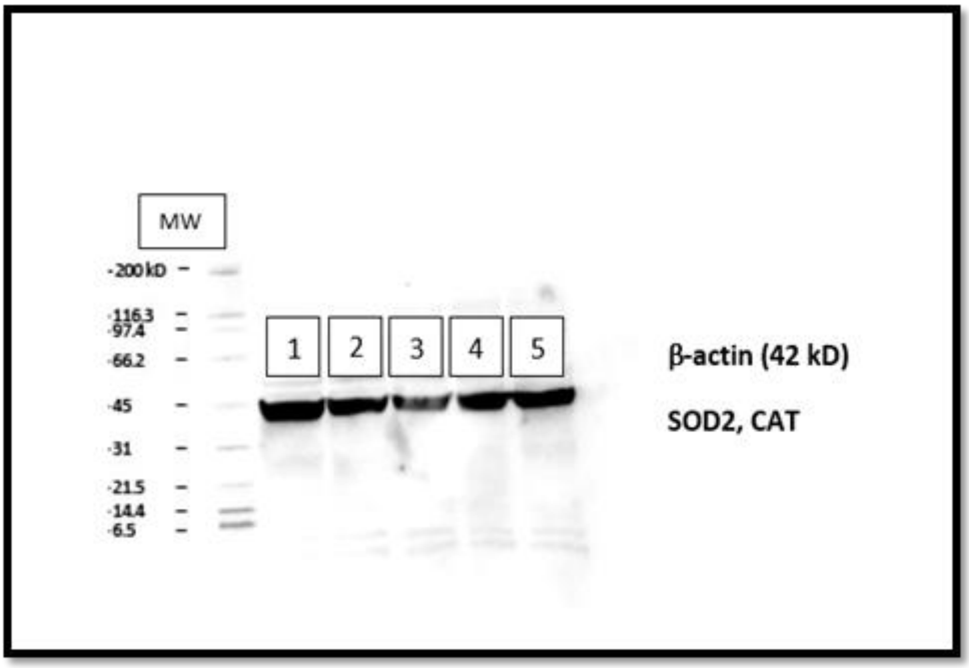

Figure S6. Western blot membrane of Beta-Actin for SOD2 and Catalase (~42 kDa). Protein detected with HRP-conjugated antibody for β-actin (A3854, Sigma-Aldrich). Densitometry readings were calculated using Image Lab™ Software v6.0 (Bio-Rad, Hercules, CA, USA). ([MW-Molecular Weight marker]; [1- Control]; [2- TDF]; [3- 3TC]; [4- DTG]; [5- TDF+3TC+DTG]).

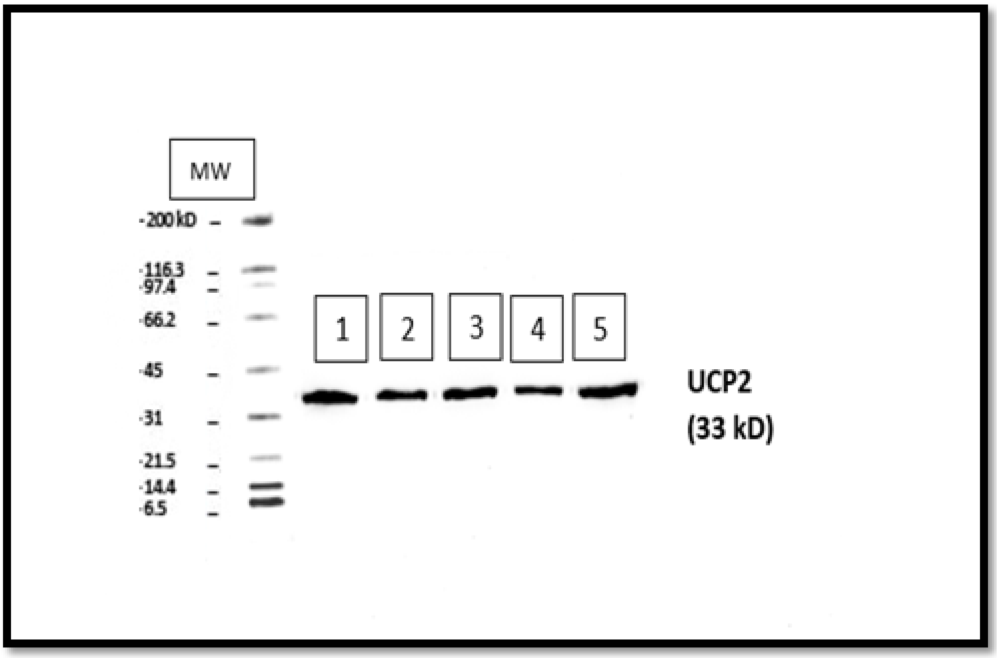

Figure S7. Western blot membrane of UCP2 (~33 kDa). Protein detected with UCP2 (D1O5V) Rabbit mAb (Cell signalling Technology; #89326S). Membranes were incubated with HRP-conjugated secondary antibody (Cell signalling Technology; anti-rabbit (#7074S)). Densitometry readings were calculated using Image Lab™ Software v6.0 (Bio-Rad, Hercules, CA, USA). ([MW-Molecular Weight marker]; [1- Control]; [2- TDF]; [3- 3TC]; [4- DTG]; [5- TDF+3TC+DTG]).

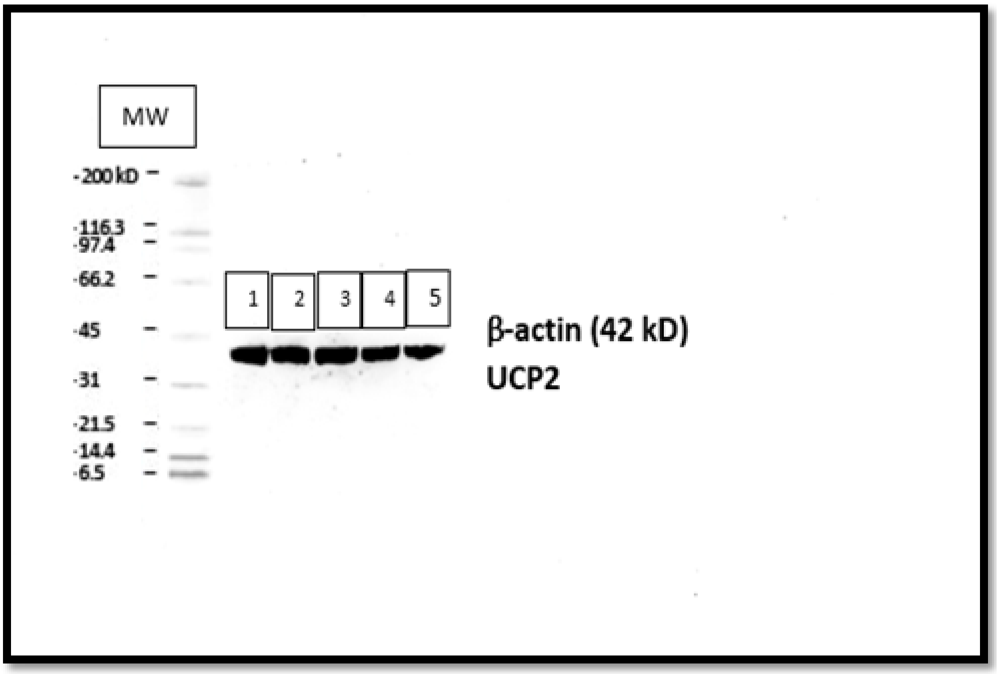

Figure S8. Western blot membrane of Beta-Actin for UCP2 (~42kDa). Protein detected with HRP-conjugated antibody for β-actin (A3854, Sigma-Aldrich). Densitometry readings were calculated using Image Lab™ Software v6.0 (Bio-Rad, Hercules, CA, USA). ([MW-Molecular Weight marker]; [1-Control]; [2- TDF]; [3- 3TC]; [4- DTG]; [5- TDF+3TC+DTG]).

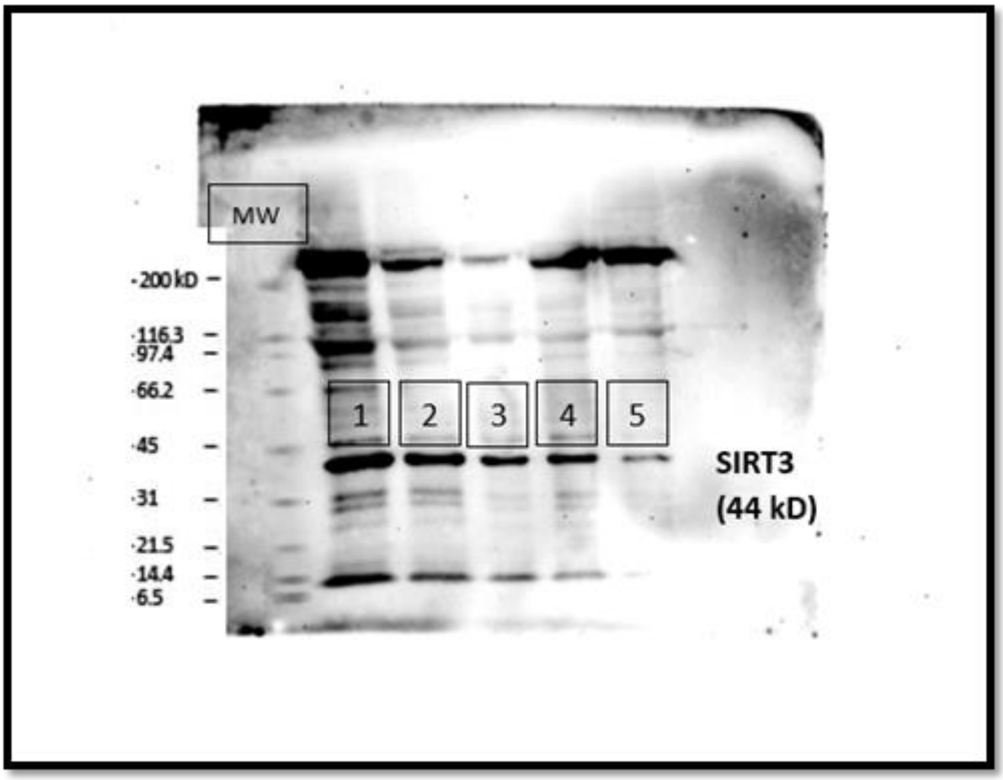

Figure S9. Western blot membrane of SIRT3 (~44kDa). Protein detected Anti-SIRT3 (Abcam; ab264041). Membranes were incubated with HRP-conjugated secondary antibody (Cell signalling Technology; anti-rabbit (#7074S)). Densitometry readings were calculated using Image Lab™ Software v6.0 (Bio-Rad, Hercules, CA, USA). ([MW-Molecular Weight marker]; [1- Control]; [2- TDF]; [3- 3TC]; [4- DTG]; [5- TDF+3TC+DTG]).

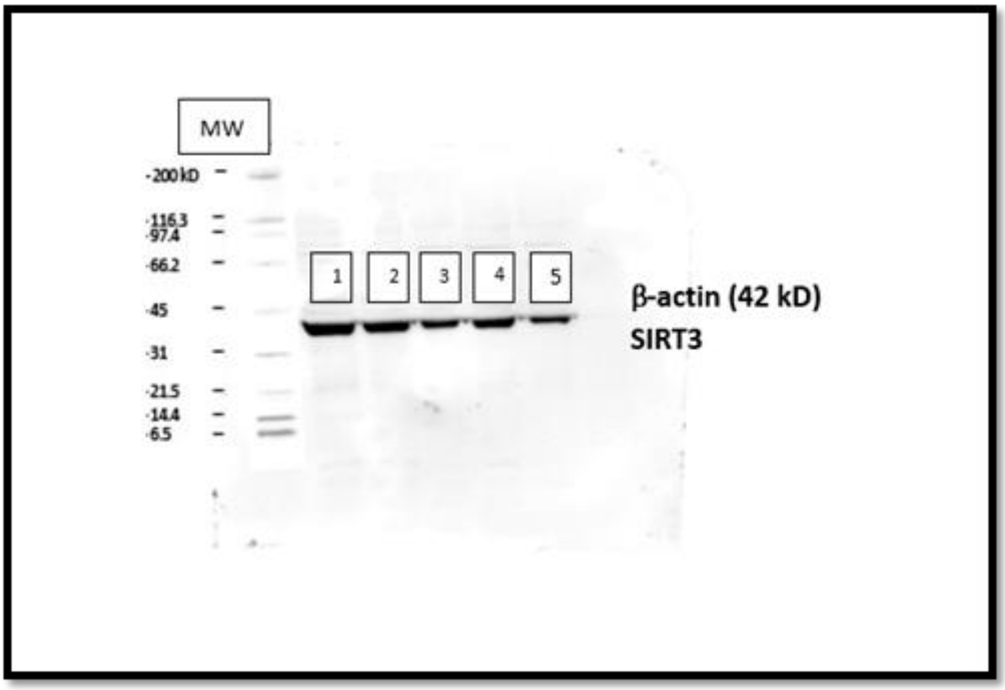

Figure S10. Western blot membrane of Beta-Actin for SIRT3 (~42kDa). Protein detected with HRP-conjugated antibody for β-actin (A3854, Sigma-Aldrich). Densitometry readings were calculated using Image Lab™ Software v6.0 (Bio-Rad, Hercules, CA, USA). ([MW-Molecular Weight marker]; [1- Control]; [2- TDF]; [3- 3TC]; [4- DTG]; [5- TDF+3TC+DTG]).

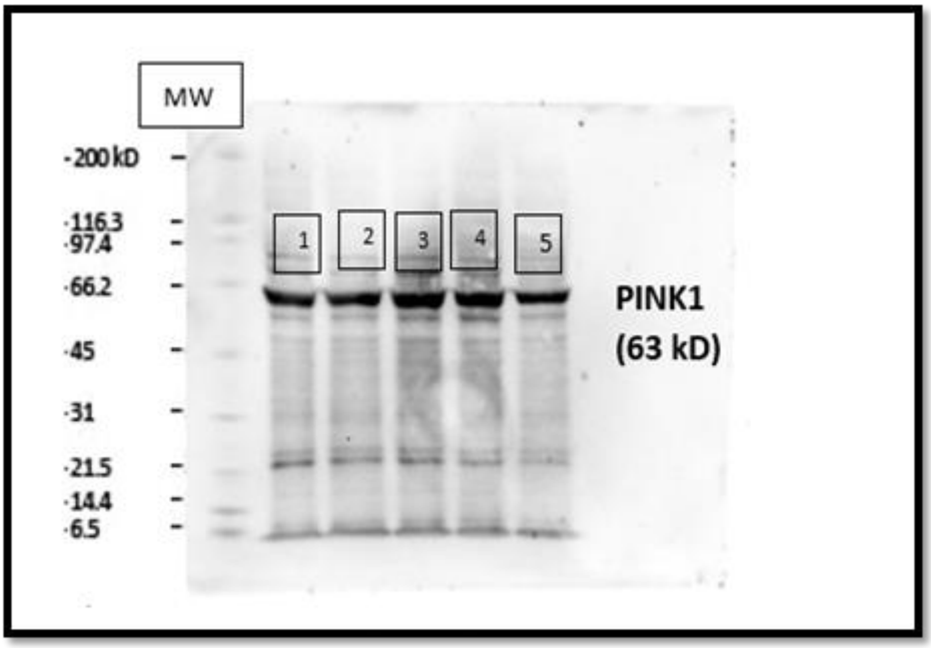

Figure S11. Western blot membrane of PINK1 (~63 kDa). Protein detected Anti-PINK1 antibody [N4/15] (Abcam; ab186303). Membranes were incubated with HRP-conjugated secondary antibody (Cell signalling Technology; anti-mouse (#7076S)). Densitometry readings were calculated using Image Lab™ Software v6.0 (Bio-Rad, Hercules, CA, USA). ([MW-Molecular Weight marker]; [1- Control]; [2- TDF]; [3- 3TC]; [4- DTG]; [5- TDF+3TC+DTG]).

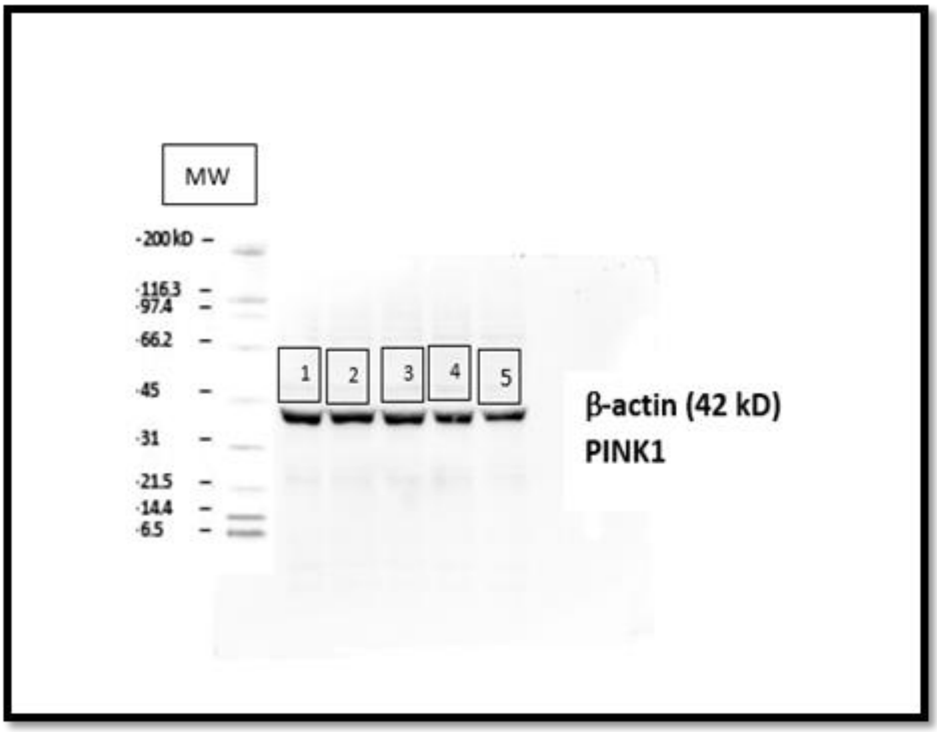

Figure S12. Western blot membrane of Beta-Actin for PINK1 (~42kDa). Protein detected with HRP-conjugated antibody for β-actin (A3854, Sigma-Aldrich). Densitometry readings were calculated using Image Lab™ Software v6.0 (Bio-Rad, Hercules, CA, USA). ([MW-Molecular Weight marker]; [1-Control]; [2- TDF]; [3- 3TC]; [4- DTG]; [5- TDF+3TC+DTG])
